# Isolation, Characterization, and Identification of Biological Control Agent for Potato Soft Rot in Bangladesh

**DOI:** 10.1100/2012/723293

**Published:** 2012-05-03

**Authors:** M. M. Rahman, M. E. Ali, A. A. Khan, A. M. Akanda, Md. Kamal Uddin, U. Hashim, S. B. Abd Hamid

**Affiliations:** ^1^Department of Plant Pathology, Bangabadhu Sheikh Mujibur Rahman Agricultural University, Gazipur 1706, Bangladesh; ^2^Nanotechnology and Catalysis Research Center, Universiti Malaya, 50603 Kuala Lumpur, Malaysia; ^3^Institute of Tropical Agriculture, Faculty of Agriculture, Universiti Putra Malaysia, 43400 Serdang, Malaysia; ^4^Institute of Nano Electronic Engineering, Universiti Malaysia Perlis, 01000 Kangar, Malaysia

## Abstract

A total of 91 isolates of probable antagonistic bacteria of potato soft rot bacterium *Erwinia carotovora* subsp. *carotovora* (Ecc) were extracted from rhizospheres and endophytes of various crop plants, different soil varieties, and atmospheres in the potato farming areas of Bangladesh. Antibacterial activity of the isolated probable antagonistic bacteria was tested *in vitro* against the previously identified most common and most virulent soft rot causing bacterial strain Ecc P-138. Only two isolates E-45 and E-65 significantly inhibited the *in vitro* growth of Ecc P-138. Physiological, biochemical, and carbon source utilization tests identified isolate E-65 as a member of the genus *Bacillus* and the isolate E-45 as *Lactobacillus* sp. The stronger antagonistic activity against Ecc P-138 was found in E-65 *in vitro* screening and storage potatoes. E-65 reduced the soft rot infection to 22-week storage potatoes of different varieties by 32.5–62.5% in model experiment, demonstrating its strong potential to be used as an effective biological control agent for the major pectolytic bacteria Ecc. The highest (62.5%) antagonistic effect of E-65 was observed in the Granola and the lowest (32.7%) of that was found in the Cardinal varieties of the Bangladeshi potatoes. The findings suggest that isolate E-65 could be exploited as a biocontrol agent for potato tubers.

## 1. Introduction

 Bacterial soft rot is one of the major postharvest diseases of potato (*Solanum tuberosum*), the fourth major food crop of the world after rice (*Oryza sativa*), maize (*Zea mays*), and wheat (*Triticum aestivum*) [1]. It is the third major food crop of Bangladesh after rice and wheat. Approximately 22% of potatoes are lost per year due to viral, bacterial, fungal, and pest attack to potato tuber and potato plant, incurring an annual loss of over 65 million tones and bacterial soft rot alone accounts for 30–50% of this huge loss [1]. The effect of the disease is more pronounced in the countries where appropriate storage facilities are lacking or inadequate. Control of bacterial soft rot of vegetables is traditionally based on phytosanitary and cultural practices [2]. Use of chemicals is generally not recommended to control soft rot [2] because of the high risk of the residual effect of toxic chemicals that might be hazardous to consumers' health.

 Biological control is a potential method to control soft rot disease [3]. The strategy for biological control of plant diseases involves the use of antagonistic microorganisms before or after the infection takes place. Commercial biological control agents are available for the seed treatments and soil amendments to protect the plants against soil borne pathogens. Currently, the bacteria *Bacillus subtilis *and *Pseudomonas *spp. and the fungi *Gliocladium virens *and *Trichoderma *spp. are the organisms mostly used for biological control strategies. Potentiality of biological control of bacterial soft rot with antagonistic bacteria or with growth promoting rhizobacteria, fluorescent pseudomonads, and endophytic bacteria in many crops has already been proven [2–5]. Potatoes could be treated with bacteria, originally selected from potato surfaces, which are inhibitory to the growth of *E. carotovora.* Fluorescent pseudomonads are attractive candidates for biological control of *E. carotovora *because they colonize the potato rhizosplane and rhizosphere and produce high populations [6–9] and they produce a variety of secondary metabolites and substances that can alter the composition of the rhizosphere microflora [10, 11]. Suppression of soft rot bacteria was attributed to the production of fluorescent siderophores that were essential for uptake of iron by the pseudomonads [12]. The bioagent *B. subtilis* was the most effective in reducing the soft rot decaying stored potato tubers [13].

 The importance of environment friendly plant protection methods is greatly emphasized in the sustainable agriculture. The recent increase in publication on bacterial endophytes reflects an interest in their potential benefits as biocontrol agents in agriculture [14]. So the development of suitable and environment friendly control measures against soft rot causing bacteria may minimize the loss in storage and improve the quality of potato. In Bangladesh, suitable measures to control soft rot of potato are yet to be developed. As the performance of an antagonistic bacterium is dependent on environment, the identification of a suitable bacterial strain which has appropriate antagonistic properties to the common potato soft rot of Bangladesh should play a significant role to develop an environmentally friendly biological control measure to prevent potato damage in Bangladesh. Considering the above facts, we extensively searched for an effective biological control agent for the most common potato soft rot strain Ecc P-138 of Bangladesh. The antagonistic activity against soft rot of potato strain Ecc P-138 was tested in *in vitro* and storage experiments, and the strain identification was performed through physiological and biochemical identification schemes.

## 2. Experimental Section

### 2.1. Isolation of Bacteria Antagonistic to Soft Rot Ecc P-138

As a potential source of antagonistic bacteria of potato soft rot Ecc, rhizospheres and endophytes of different crop plants as well soils and atmosphere of major potato farming areas in Bangladesh were selected. For the isolation of rhizospheric bacteria, soil samples were collected from rhizosphere of potato, onion, papaya, rice, tomato, garlic, zinger, and turmeric. Dilution plate techniques were followed, and yeast peptone dextrose agar (YPDA) was used as a basic medium. Ten gram (10 g) of soil was taken from each rhizosphere soil samples in a beaker and was mixed thoroughly in 100 mL distilled water on a rotary shaker (250 rpm). Then, the suspension was allowed to settle down to soil for 10 min. After sedimentation, the soil suspension was taken from the upper part and diluted to 10^3^–10^4^ x in distilled water. The diluted soil suspension was streaked on petri dishes containing YPDA and incubated at room temperature for 24–48 h. At the end of the incubation, different types of bacterial colonies that appeared on the medium were selected and restreaked for pure culture. The pure culture of selected bacterial isolates was preserved in test tubes containing sterilized water for antibacterial evaluation.

 To isolate endophytic bacteria, fresh and diseased plant specimens of root, stem, and leaves of various plants including potatoes and onions were collected from different locations of Bangladesh. Desired bacteria were isolated following streak-plate technique using the medium YPDA. After 24–48 h of incubation, bacterial colonies were observed on the medium. Isolated bacterial colonies were restreaked on YPDA media for obtaining pure culture. Colonies selected from isolated plates were transferred into test tubes containing 5 mL sterilized distilled water. The tubes with the bacterial suspension were preserved at room temperature.

 Some bacterial isolates were isolated from soil mixed with compost following the procedures as described in case of rhizospheric bacteria. Several bacteria were isolated from atmosphere following air trapping. Three petri dishes containing YPDA were placed in the field and also in the laboratory of Bangabandhu Sheikh Mujibor Rahman University (BSMRAU) and were kept for 5, 10, and 15 min. The petri dishes were covered with the lids and incubated at room temperature for 24–48 h. The bacterial colonies developed on the medium were restreaked for pure culture and preserved in test tubes containing sterilized distilled water.

### 2.2. Antagonistic Activity Test

 Antagonistic activity of the bacterial isolates was tested *in vitro *using plate chloroform method [15, 16]. Briefly, one loop full of 1-2-day-old probable antagonistic bacterial colony grown in YPDA medium was transferred to the center of a petri dish containing 20 mL of YPDA. The plates were incubated at 30°C for 2 to 3 days. When the bacteria colonies were formed as several millimeters in diameter, the plate was turned upside down. A sheet of filter paper was placed in the petri dish lid with 0.5 mL of chloroform. The dish was kept at room temperature for 2 h. After complete evaporation of chloroform vapor, 5 mL suspension of indicator bacteria (ca. 10^8^ cfu/mL) was overlaid on each plate. Here, the soft rot bacterial strain, *E. carotovora *subsp*. carotovora *(Ecc) P-138, was used as an indicator bacterium. The plate thus prepared was incubated at 30°C for 2 days. When an inhibition zone appeared, its diameter was measured to evaluate the antibacterial activity of the probable antagonistic bacteria [16]. The bacterial isolates which showed antagonistic effect against indicator bacteria were selected for the further studies.

### 2.3. Biological Control of Soft Rot Disease under Storage Conditions

To evaluate the effectiveness of the selected antagonistic bacteria in reducing soft rot infection in storage potatoes, 700 g of fresh tubers of each three potato varieties, Cardinal, Diamant, and Granola, were dipped in suspensions of antagonistic bacterium E-65 (ca. of 10^7^–10^8^ cfu/mL), for 30 min and air-dried separately. The treated potato tubers were inoculated with soft rot bacteria *E. carotovora *subsp*. carotovora *P-138 by spraying with inoculum suspensions (10^7^–10^8^ cfu/mL) with an atomizer. Inoculated potato tubers bulbs were air-dried and stored separately in net bags at room temperature. Data on soft rot incidence was recorded after 2, 6, 10, 14, 18, and 22 weeks of inoculation. Number and weight of soft rot infected tubers were recorded and expressed in percentage using the following formula described by Abd- El-Khair and Karima [13]:

(1) $$\begin{array}{cc} & \text{Infection}\;\text{\%}\\ & =\frac{\text{number}\;\text{of}\;\text{infected}\;\text{tubers}}{\text{Total}\;\text{number}\;\text{of}\;\text{tubers}}\times 100,\\ & \text{Loss}\;\text{of}\;\text{weight}\;\text{\%}\\ & \;=\frac{\text{Initial}\;\text{weight}-\text{weight}\;\text{after}\;\text{discarding}\;\text{the}\;\text{infected}\;\text{sample}}{\text{Initial}\;\text{weight}}\\ & \;\times 100. \end{array}$$

Percentage of disease reduction (PDR) was calculated according to the following formula described by Hajhamed et al. [17]:

(2) $$\begin{array}{c} \text{PDR}=\frac{\text{Ack}-\text{Atr}}{\text{Ack}}\times 100 \end{array},$$

where Ack and Atr represent the severity of the disease in control and treated specimens, respectively.

### 2.4. Characterization and Identification of Antagonistic Bacterial Strains

Preliminary characterization of the selected antagonistic bacterial isolates was performed by a series of physiological and biochemical tests as described earlier. The tests were potato soft rot [18], gram reaction [19], growth at 37°C, growth in 5% NaCl [20, 21] catalase production [22], oxidase reaction [23], nitrate reduction [22], methyl red test [21], arginine utilization test [24], gas formation test [25], levan formation test [26], and tobacco hypersensitivity reaction (HR) test [27]. Carbon source utilization tests were performed using Ayer's media [28].

## 3. Results

### 3.1. Antagonistic Test

Out of 91 probable antagonistic bacterial isolates, 28 isolates were extracted from rhizosphere soil of nine crops: potato, onion, papaya, rice, tomato, garlic, zinger, and turmeric collected from Rangpur, BSMRAU, and Bangladesh Agricultural Research Institute (BARI) located at Gazipur. The 44 endophytic bacterial isolates were obtained from 13 plant varieties; 4 isolates were from compost fertilizers, 3 isolates were from atmosphere, and 12 isolates were from microbiology laboratory of BSMRAU. The host, host organs, locations and time of isolation, media used for isolation, and colony characters are shown in Tables 1–6 (See Supplementary Information available online at doi: 10.1100/2012/723293). 

### 3.2. Selection of Antagonistic Strain of the Most Common Potato Soft Rot Bacterium

**Figure 1:**
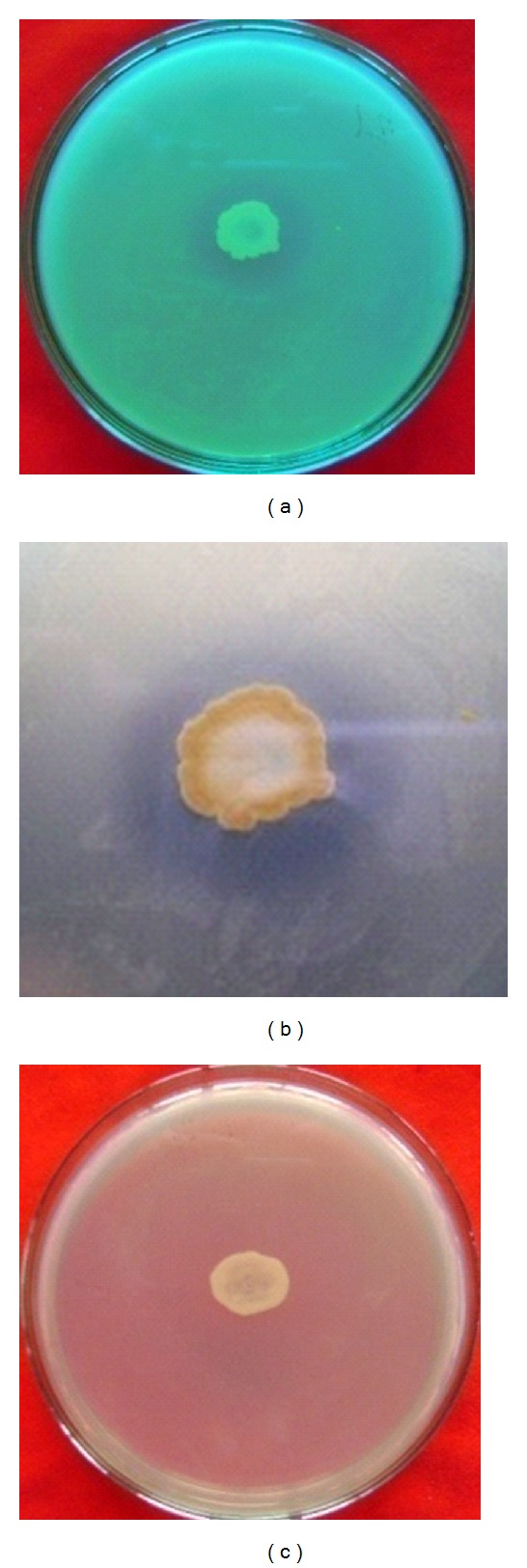
Antagonistic activity of isolates E-65 showing inhibition zones against potato soft rot bacterial strain Ecc P-138. (a) and (b) are representatives of positive inhibition as shown by the encircled inhibition zones, and (c) is presenting negative inhibition as demonstrated by the no-inhibition zone.

Among the 91 bacterial isolates tested for antagonistic activity, only 2 of them (isolates E-65 and E-45) demonstrated significant antagonistic effects against the indicator soft rot Ecc P-138 (Tables 1–6). Isolates E-45 and E-65 were extracted from the endophytes of papaya and potato. These bacterial isolates produced distinct inhibition zones around their respective colonies (Figure 1). The diameter of the inhibition zones around the antagonistic bacterial colonies E-45 and E-65 was 4 ± 1.1 mm and 10 ± 1.85 mm in triplicates *in vitro *experiments indicating stronger antagonistic actions of E-45 against the soft rot potato bacterium Ecc P-138 than that of E-45. Therefore, the isolate E-65 was selected for the control trial of storage potatoes against the most common soft rot bacterial strain Ecc P-138.

### 3.3. The Effect of Antagonistic Bacterial Strain E-65 on Storage Potato Tubers

**Figure 2:**
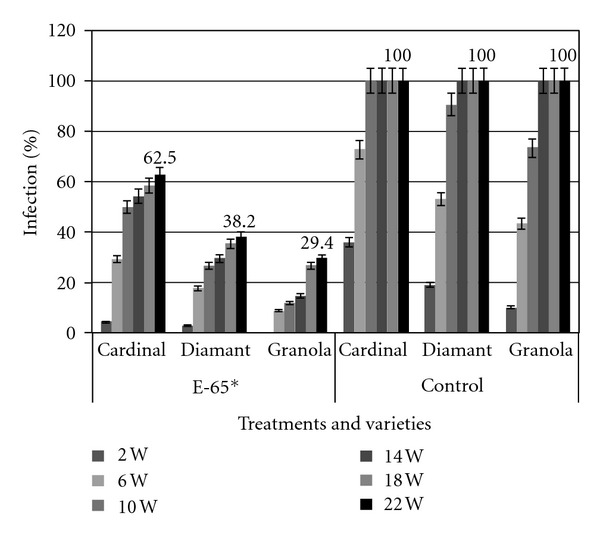
Effect of antagonistic bacteria* (E-65) on soft rot incidence of potato in storage as observed at an interval of 4 weeks (W).

The incidence of the soft rot bacterial infection to the potato tubers of different varieties at different time of intervals is shown in Figure 2. The infection started from the second week or later from the inoculation of soft rot bacterium Ecc P-138 and treatment with antagonistic bacterium (E-65). In the Cardinal and Diamant varieties, the infection was recorded at the second week, but, in the Granola variety, no infection was observed within the same period. After 22 weeks of storage, all varieties were infected. However, the highest infection (62.5%) was observed in Cardinal variety and the lowest rate of infection (29.4%) was found in the Granola variety. The Diamant variety demonstrated 38.2% infection after 22 weeks. On the other hand, the control group or E-65 untreated potatoes of all varieties incurred 100% damages within 22 weeks of the experimental time (Figure 2).

**Figure 3:**
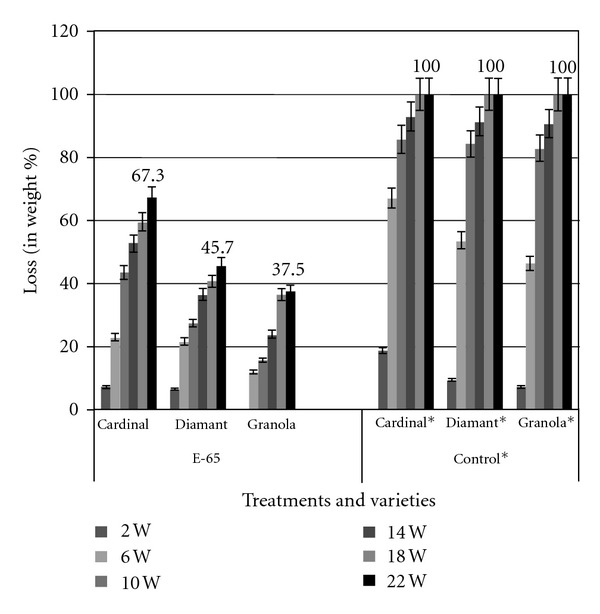
Effect of antagonistic bacteria (E-65) on weight loss (% w/w) of three potato varieties under storage conditions.

**Figure 4:**
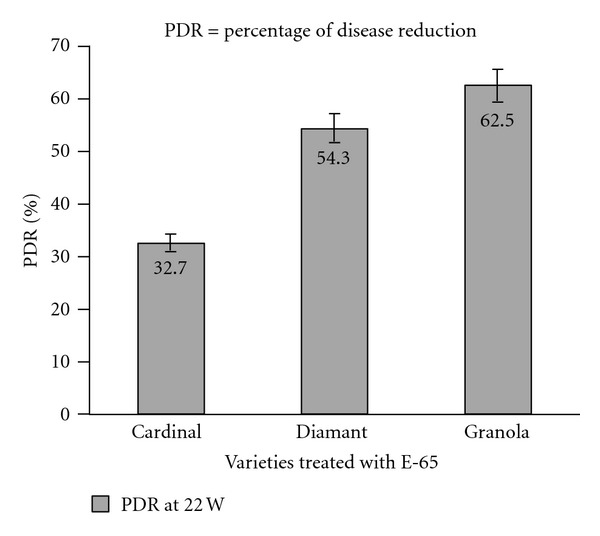
Effect of antagonistic bacteria (E-65) on the percentage of soft rot disease reduction (PDR) of potato at 22 weeks (W) of storage.

 The effects of antagonistic bacteria on the weight loss (% w/w) of potato tubers due to soft rot infection are shown in Figure 3. At the 22nd week of storage, the weight loss due to soft rot infection ranged 37.5–67.3% in different varieties under antagonistic bacteria treatment, while, in control group the loss was 100%. The lowest loss was recorded in the variety Granola (37.5%) and highest in Cardinal (67.3%). The weight losses were always higher in control group. The highest reduction of disease due to treatment with antagonistic bacteria was found in Granola (62.5%), and the lowest was observed in Cardinal (32.7%) varieties (Figure 4). Thus, the Granola variety performed the best against the soft rot strain Ecc P-138 which was followed by Diamant and Cardinal.

### 3.4. Characterization and Identification of Two Antagonistic Bacterial Isolates

Two antagonistic bacterial isolates were Gram-positive and -negative in catalase, potato soft rot, and oxidase tests. The isolate E-45 grew weakly in both at 37°C and 6.5% NaCl. The isolate E-65 grew well in 6.5% NaCl but weakly at 37°C. In nitrate reduction test, isolate E-65 showed weak positive reaction with the development of orange-brown color, but isolate E-45 showed clear positive reaction. Two isolates showed negative reaction in arginine utilization test. No gas formation was found in case of isolate E-65. Two isolates were negative in production of fluorescent pigment on King's B medium and tobacco hypersensitivity test (Table 4 Supplementary Information).

 Isolate E-65 did not utilize cellobiose, lactose, maltose, L-arabinose, D-galactose, D-xylose, raffinose, sucrose, and trehalose but was positive in benzoate and D-tartrate. While the isolate E-45 utilized cellobiose, maltose, L-arabinose, D-galactose, D-xylose, raffinose, and sucrose but was negative in lactose, benzoate, and D-tartrate (Tables 5 and 6 Supplementary Information).

On the basis of the above physiological, biochemical, and carbon sources utilization test results, the antagonistic bacterial strain E-65 was identified as a member of the genus *Bacillus* and the isolate E-45 was a member of *Lactobacillus* sp. However, comprehensive molecular identification is necessary for assignment of the exact species of the isolated strains.

## 4. Discussion

The results of the present study demonstrated that the identified antagonistic bacterial strain E-65 can significantly inhibit the growth of soft rot bacteria *in vitro* and in storage. The pretreatment of potato tubers with antagonistic bacteria successfully prevented the initial infection and reduce soft rot disease of potato and multiplication of soft rot bacteria. Earlier studies reported that antagonistic endophytic and rhizospheric bacteria have significant antagonistic activity against plant pathogenic bacteria including soft rot *Erwinia *genera [13, 29–32]. Long et al. [29] reported that the genus *Bacillus* and fluorescent pseudomonads have antagonistic activity against various plant pathogenic bacteria including soft rot bacterium *E. carotovora* subsp. *carotovora *(Ecc) *in vitro*. The ability of these isolates to suppress the growth of various phytopathogenic bacteria makes them potential biocontrol agents.

 In the present study, biochemical and physiological identification methods suggested that identified antagonistic bacterial strain was *Bacillus *sp. and demonstrated strong antagonistic actions against the soft rot bacterial pathogen Ecc P-138 which was previously identified in our lab as the most virulent strain of* Erwinia carotovora *subsp*. carotovora* both in *in vitro* and under storage conditions. Many researchers previously exploited *Bacillus *sp. to control soft rot bacteria in various plants [13, 30–32]. The results of the present experimental findings strongly correlated with those of the earlier findings [13, 30, 32]. Thus, the pretreatment of potato tubers with the identified biocontrol-agents can retard the initial infection of soft rot disease and inhibit the multiplication of soft rot pathogen. However, the present study was performed with 700 g potato tubers which are not sufficient to recommend its application for the large-scale storage of the potato tubers. The efficacy of the antagonistic bacteria needs to be tested using large amount of tubers before recommendation.

## 5. Conclusion

 Two antagonistic bacterial isolates E-65 and E-45 of the common soft rot bacterial pathogen were isolated from the endophytes of potato and papaya of Bangladesh. The isolate E-65 was a strong antagonist of soft rot bacteria *E. carotovora *subsp*. carotovora* P-138. This was confirmed through the *in vitro* and storage experiments. Biochemical and physiological tests identified E-65 as a *Bacillus* sp. and E-45 as a* Lactobacillus* sp. The ability of these isolates to suppress the growth of various phytopathogenic bacteria makes them potential biocontrol agents.

## Supplementary Material

A total of 31 bacterial strains, designated as R-1 to R-31, were isolated from the rhizosphere of various plants and described in table 1. None of this strain demonstrated antagonistic activity against the soft soft rot causing bacterial strain P-138. A total of 48 bacterial strains, designated as E-35 to E-82, were extracted from the endophytes of 19 different plant species from 5 different potato-farming areas of Bangladesh and presented in table 2. Only the isolates E-45 and E-65 demonstrated antagonistic activity in *in vitro* test. A total of 4, 3 and 12 bacterial strains were isolated from compost fertilizer (C-28-C-31), atmosphere (A-59- A-61) and BSMRAU laboratory (L-85-L-96) and are preseted in table 3. None of these bacterial isolates demonstrated antagonistic activity against the soft soft rot causing bacterial strain P-138. The species identification schemes of isolates E-45 and E-65 using various physiological and biochemical tests are presented in tables 4-6. The combined sets of identification tests strongly suggested that antagonistic function containg isolates E-45 and E-65 were belonged to Lactobacillus and Bacillus species, respectively.
