# Supplementary material for: Isolation, Characterization, and Identification of Biological Control Agent for Potato Soft Rot in Bangladesh
Source: ScientificWorldJournal. 2012 May 3;2012:723293. doi: 10.1100/2012/723293 (PMC3356727; doi:10.1100/2012/723293)
Supplement: Supplementary file 1 — A total of 31 bacterial strains, designated as R-1 to R-31, were isolated from the rhizosphere of various plants and described in table 1. None of this strain demonstrated antagonistic activity against the soft soft rot causing bacterial strain P-138. A total of 48 bacterial strains, designated as E-35 to E-82, were extracted from the endophytes of 19 different plant species from 5 different potato-farming areas of Bangladesh and presented in table 2. Only the isolates E-45 and E-65 demonstrated antagonistic activity in in vitro test. A total of 4, 3 and 12 bacterial strains were isolated from compost fertilizer (C-28-C-31), atmosphere (A-59- A-61) and BSMRAU laboratory (L-85-L-96) and are preseted in table 3. None of these bacterial isolates demonstrated antagonistic activity against the soft soft rot causing bacterial strain P-138. The species identification schemes of isolates E-45 and E-65 using various physiological and biochemical tests are presented in tables 4-6. The combined sets of identification tests strongly suggested that antagonistic function containg isolates E-45 and E-65 were belonged to Lactobacillus and Bacillus species, respectively. [file 723293.f1.pdf]

Table 1 List of probable antagonistic bacteria isolated from rhizosphere of various crops and tested for antagonistic activity against soft rot bacteria of potato

Isolates No.	Sources (Rhizosphere)	Locations	Colony Characters on YPDA	Antagonistic activity
R-1	Papaya	Rangpur	Yellow	-
R-2	,,	,,	Creamy white	-
R-3	Rice	,,	White	-
R-4	,,	,,	Creamy white	-
R-5	,,	,,	White	-
R-6	Potato	,,	White dry	-
R-7	,,	,,	,,	-
R-8	,,	,,	White sticky	-
R-9	,,	,,	White	-
R-10	,,	,,	,,	-
R-11	,,	,,	Creamy white sticky	-
R-12	,,	,,	Very small white	-
R-13	,,	,,	White	-
R-14	,,	BSMRAU	White big	-
R-15	,,	,,	White	-
R-16	,,	,,	White sticky	-
R-17	,,	BARI	Yellow white	-
R-18	,,	,,	White smal	-
R-19	,,	,,	White big	-
R-20	Tomato	,,	White	-
R-21	,,	,,	Yellow	-
R-22	,,	,,	Creamy white	-
R-23	Onion	,,	White big	-
R-24	,,	BSMRAU	White	-
R-25	Garlic	BARI	White big	-
R-26	Zinger	,,	White	-
R-27	Turmeric	,,	White big	-
R-31	Tomato	BSMRAU	White big	-

BARI= Bangladesh Agricultural Research Institute

Table 2 List of probable antagonistic bacteria isolated from endophytes of various crops and tested for antagonistic activity against soft rot bacteria of potato

Isolates No.	Sources/(Endophytes of)	Locations	Colony Characters on YPDA	Antagonistic activity
E-35	Cheerota	BSMRAU	White	-
E-36	,,	,,	Creamy white	-
E-37	Gada/Marigold	,,	White	-
E-38	,,	,,	Creamy White	-
E-39	K. begun	,,	Yellowish White	-
E-40	G. kumari	,,	Creamy White	-
E-41	,,	,,	Yellow	-
E-42	,,	,,	White	-
E-43	Kachu/Taro	,,	Yellow	-
E-44	Tomato	,,	Yellow White	-
**E-45**	Papaya	**,,**	White small	+
E-46	Onion	,,	Yellow	-
E-47	,,	,,	White	-
E-48	Garlic	,,	Creamy White	-
E-49	,,	,,	Yellowish White	-
E-50	Bilimbi	,,	Very small White	-
E-51	Onion	Pabna	White	-
E-52	,,	,,	White	-
E-53	,,	,,	White	-
E-54	,,	,,	White	-
E-55	Potato	Rangpur	White big	-
E-56	,,	,,	White big	-
E-57	,,	,,	Creamy White	-
E-62	Balsam	,,	Very small White	-
E-63	Olkachu/Elephant foot	,,	Creamy White	-
E-64	Tomato	,,	Creamy White	-
**E-65**	Potato	Gazipur	White	+
E-66	,,	,,	White	-
E-67	,,	,,	Creamy White	-
E-68	,,	,,	White	-
E-69	,,	,,	White small	-
E-70	,,	,,	,,	-
E-71	,,	,,	,,	-
E-72	Indian onion	,,	White	-
E-73	,,	,,	White	-
E-74	,,	,,	White sticky	-
E-75	,,	,,	White small	-
E-76	,,	,,	,,	-
E-77	,,	,,	White dry	-
E-78	Deshi onion	BARI	White	-
E-79	Potato	Dhaka	White	-
E-80	,,	,,	White small	-
E-81	,,	,,	White	-
E-82	,,	,,	White	-

Table 3 List of probable antagonistic bacteria isolated from compost fertilizer, atmosphere and Laboratory of BSMRAU and tested for antagonistic activity against soft rot bacteria of potato

Isolates No.	Sources	Locations	Colony Characters on YPDA	Antagonistic activity
C-28	Compost	BARI	White	-
C-29	,,	,,	White big	-
C-30	,,	,,	White	-
C-31	,,	BSMRAU	White big	-
A-59	Air trapping	,,	Redish	-
A-60	,,	,,	Creamy white	-
A-61	,,	,,	White dry	-
L-85	Lab isolate	Microbio-logy lab.	White big	-
L-86	,,	,,	Yellow big	-
L-87	,,	,,	White big	-
L-88	,,	,,	White	-
L-89	,,	,,	,,	-
L-90	,,	,,	,,	-
L-91	,,	,,	White big	-
L-92	,,	,,	,,	-
L-93	,,	,,	Very small white	-
L-94	,,	,,	White big	-
L-95	,,	,,	,,	-
L-96	,,	,,	White big	-

Table 4 Physiological and biochemical characteristics of antagonistic bacteria E-45 and E-65

Name of Tests	Antagonistic isolates		**Bacillus* sp.	**Lacto-bacillus* sp.
	E-45	E-65		
Potato soft rot	-	-	-	-
Gram reaction	+	+	+	+
Growth at 37 ^{0}C	w+	w+	+	+
Growth in 6.5% NaCl	w+	+	+	NA
Catalase	-	-	-	-
Oxidase	d+	-	-	-
Nitrate reduction	+	w+	+	+
Arginine utilization	-	-	NA	NA
Gas formation	w+	-	-	w+
Flurescent pigment on K'B	-	-	-	-
Tobacco hypersensitivity	-	-	-	-

*=According to Kreigh and Holt 1984 (Bergey's manual) and Long *et al.* (2003);
+ = growth positive; - = negative; d+=delay positive; d= doughtful; w+= weak growth; NA= Not available

Table 5 Utilization of different sugar as source of carbon by antagonistic bacteria E-45 and E-65

Name of carbon sources	Antagonistic isolates		**Bacillus* sp.	**Lactobacillus* sp.
	E-45	E-65		
Cellubiose	+	-	+/-	NA
Lactose	-	-	+/-	NA
Maltose	+	-	+/-	NA
L-Arabinose	+	-	-	+
D-Galactose	+	-	-	+
D-Xylose	+	-	+/-	NA
Raffinose	+	-	+/-	NA
Sucrose	+	-	+/-	NA
Trehalose	+	-	+/-	NA

*=According to Kreigh and Holt 1984 (Bergey's manual) and Long *et al.* (2003)
+ = growth positive; - = negative; d+=delay positive; NA= Not available

Table 6 Utilization of different alcohols and organic acids by antagonistic bacteria E-45 and E-65

Name of carbon sources	Antagonistic isolates		**Bacillus* sp.	**Lactobacillus* sp.
	E-45	E-65		
Dulcitol	d+	-	v	+/-
Inositol	+	-	-	+/-
Manitol	+	-	-	+/-
Sorbitol	+	-	-	+/-
Benzoate	-	+	+	-
D- Tartrate	-	+	+	-

*=According to Kreigh and Holt 1984 (Bergey's manual) and Long *et al.* (2003);
+ = Growth positive; - = negative; d+= delay positive; v= variable reaction;
NA= Not available
